# Supplementary figures and images for: Loquacious modulates flaviviral RNA replication in mosquito cells
Source: PLoS Pathog. 2022 Apr 28;18(4):e1010163. doi: 10.1371/journal.ppat.1010163 (PMC9089905; doi:10.1371/journal.ppat.1010163)

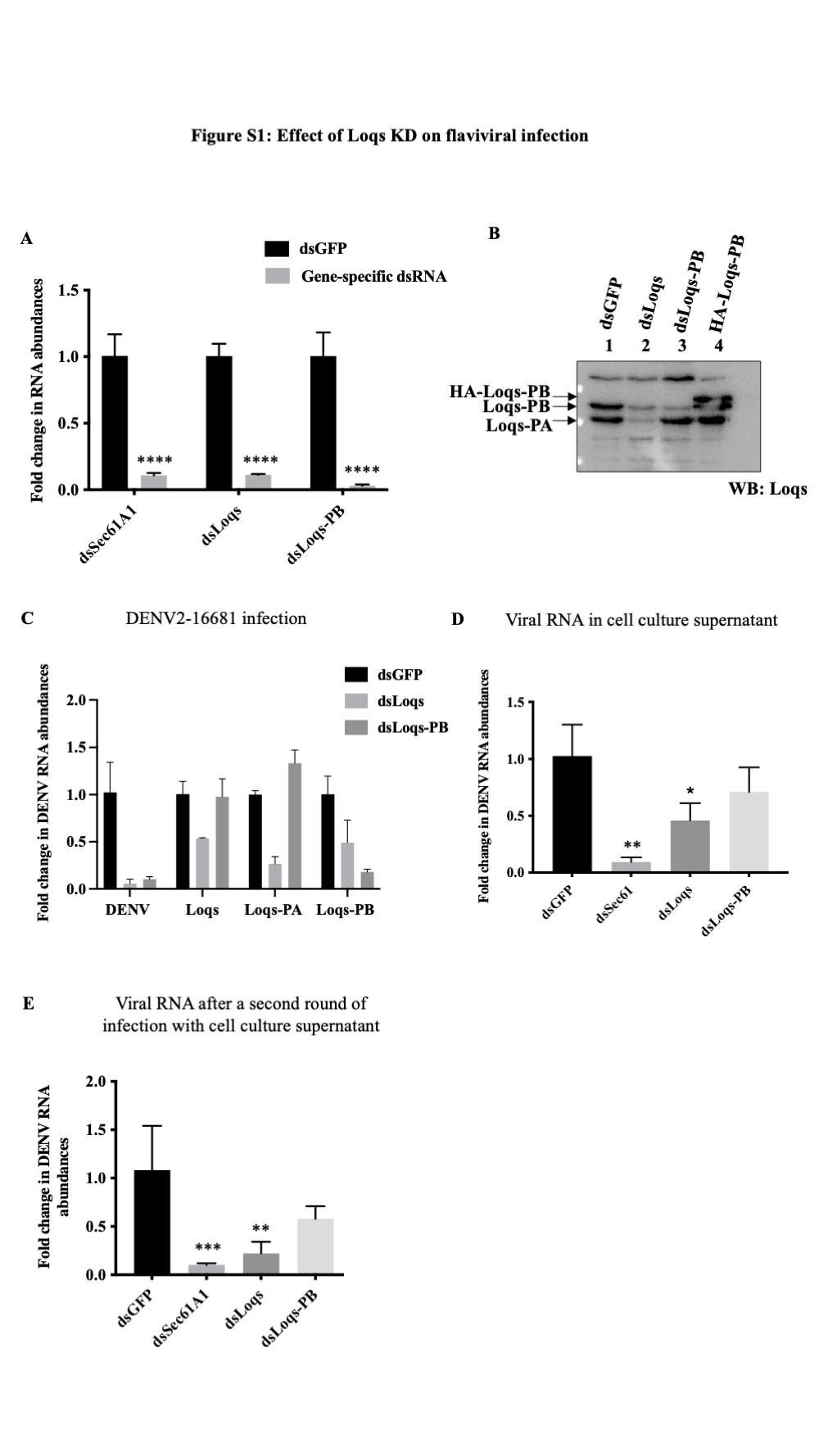

Supplement: S1 Fig — (A) RT-qPCR measurement of mRNA abundances in Aag2 cells transfected with the indicated dsRNAs. Knockdown efficiency was measured using gene-specific primers (n = 3, ****p<0.0001). (B) Western blot analysis of Loqs protein abundance in dsGFP, dsLoqs, dsLoqs-PB and HA-Loqs PB transfected Aag2 cells (C) Effects of dsRNA treatment on DENV2-16681(Thailand strain) infection of Aag2 cells (MOI = 0.1, 96 hrs), measured by RT-qPCR. Knockdown efficiency was measured using gene-specific primers. Measurements are represented as fold-change over dsGFP (n = 3, *p<0.05, **p<0.005). (D) Effect of dsRNA treatment on extracellular abundances of DENV2-16681 viral RNA in infected Aag2 cells, measured by RT-qPCR. Data is plotted as fold- change over dsGFP from three independent experiments. (E) Cell culture supernatants from dsRNA-treated cells were used to infect naive Aag2 cells and viral RNA abundances in these cells were measured by RT-qPCR (n = 3, **p<0.05, ***p<0.005). (TIF) [file ppat.1010163.s001.tif]

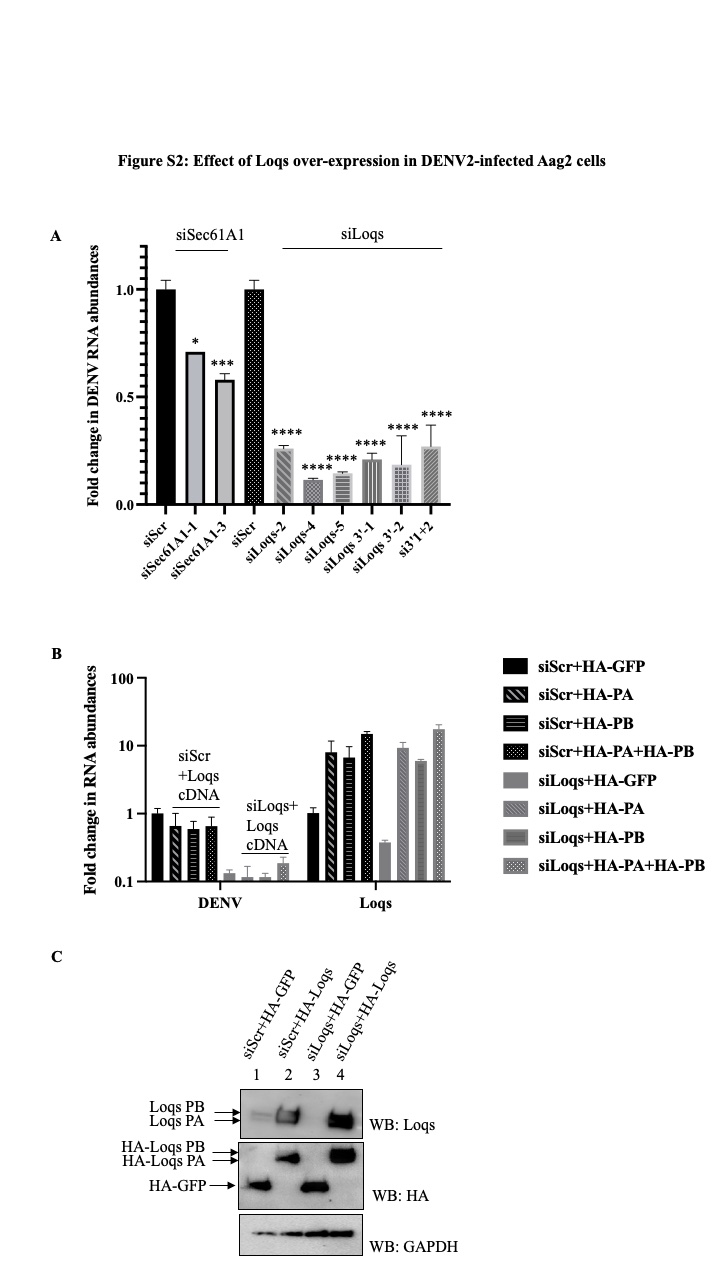

Supplement: S2 Fig — (A) Aag2 cells were transfected with the indicated siRNAs for 24 hrs followed by DENV2 infection. Cells were harvested for qPCR at 96 hrs post infection. Intracellular DENV2 RNA abundances are represented as average fold-change over siScr from three independent experiments (*p<0.05, ***p<0.0005, ****p<0.0001). (B) Aag2 cells were co-transfected with scrambled (siScr) or Loqs (siLoqs 3’-2) siRNAs and the indicated plasmid DNAs. 24 hrs post transfection they were infected with DENV2-NGC virus at a MOI of 0.1. Cells were harvested at 96 hrs post infection. Intracellular DENV2 RNA abundances were measured by RT-qPCR and are represented as average fold-change over the siScr from three independent experiments. (C) Western blot analysis of Loqs, HA-GFP, HA-Loqs-PA, HA-Loqs-PB and GAPDH protein abundances in siRNA-treated cells. (TIF) [file ppat.1010163.s002.tif]

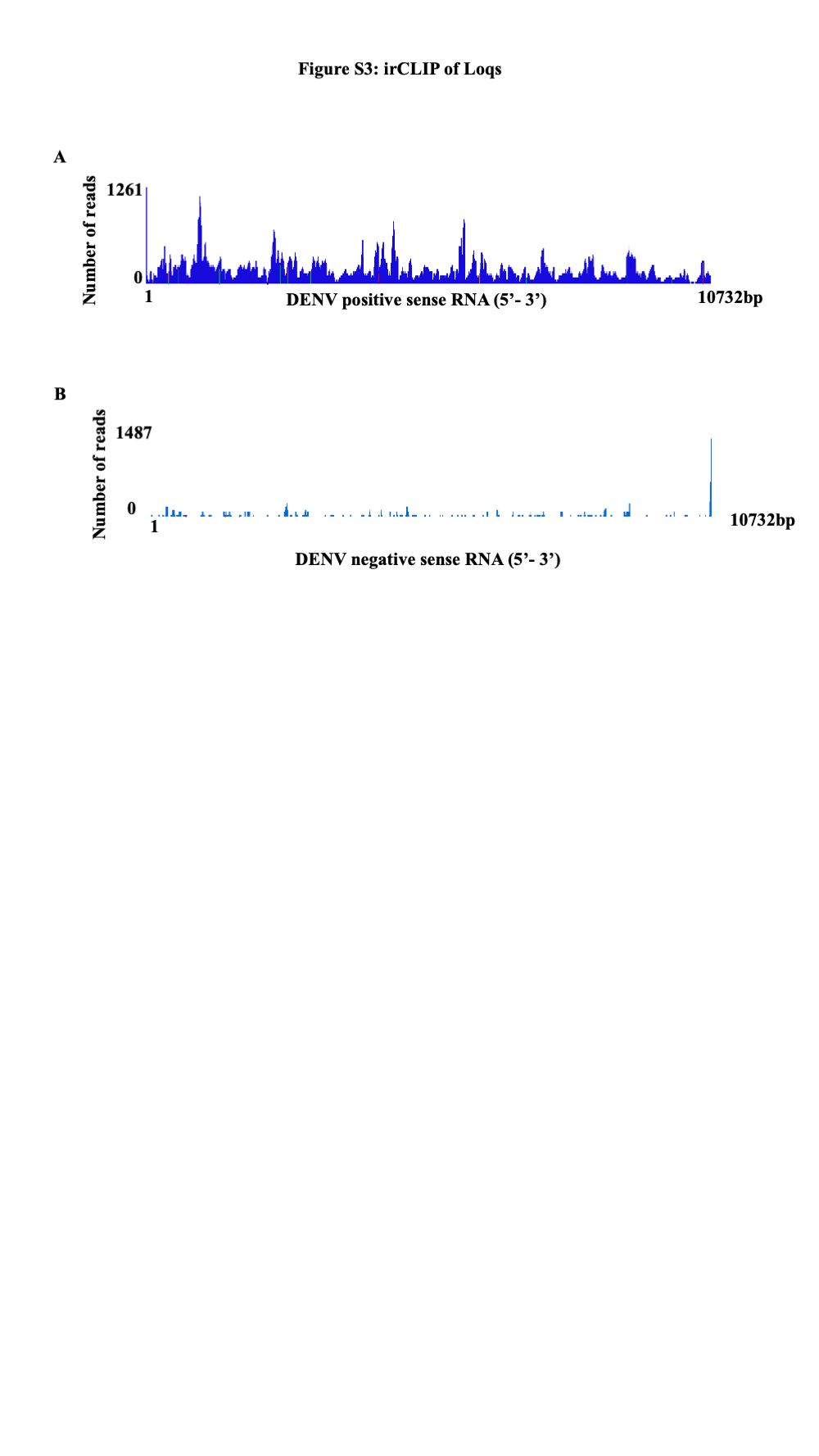

Supplement: S3 Fig — (A, B) Aag2 cells were transfected with HA-GFP or HA-Loqs PA/PB plasmids. 24 hrs post transfection, cells were infected with DENV2-NGC at a MOI of 1. Cells were UV irradiated at 254nm to covalently crosslink RNA-protein interactions and subjected to irCLIP with anti-Loqs followed by anti-HA antibodies. irCLIP RT stops were mapped at base resolution to the DENV genome. The read density across positive- (A) and negative- (B) sense DENV RNAs is represented as an average of three independent experiments. (TIF) [file ppat.1010163.s003.tif]

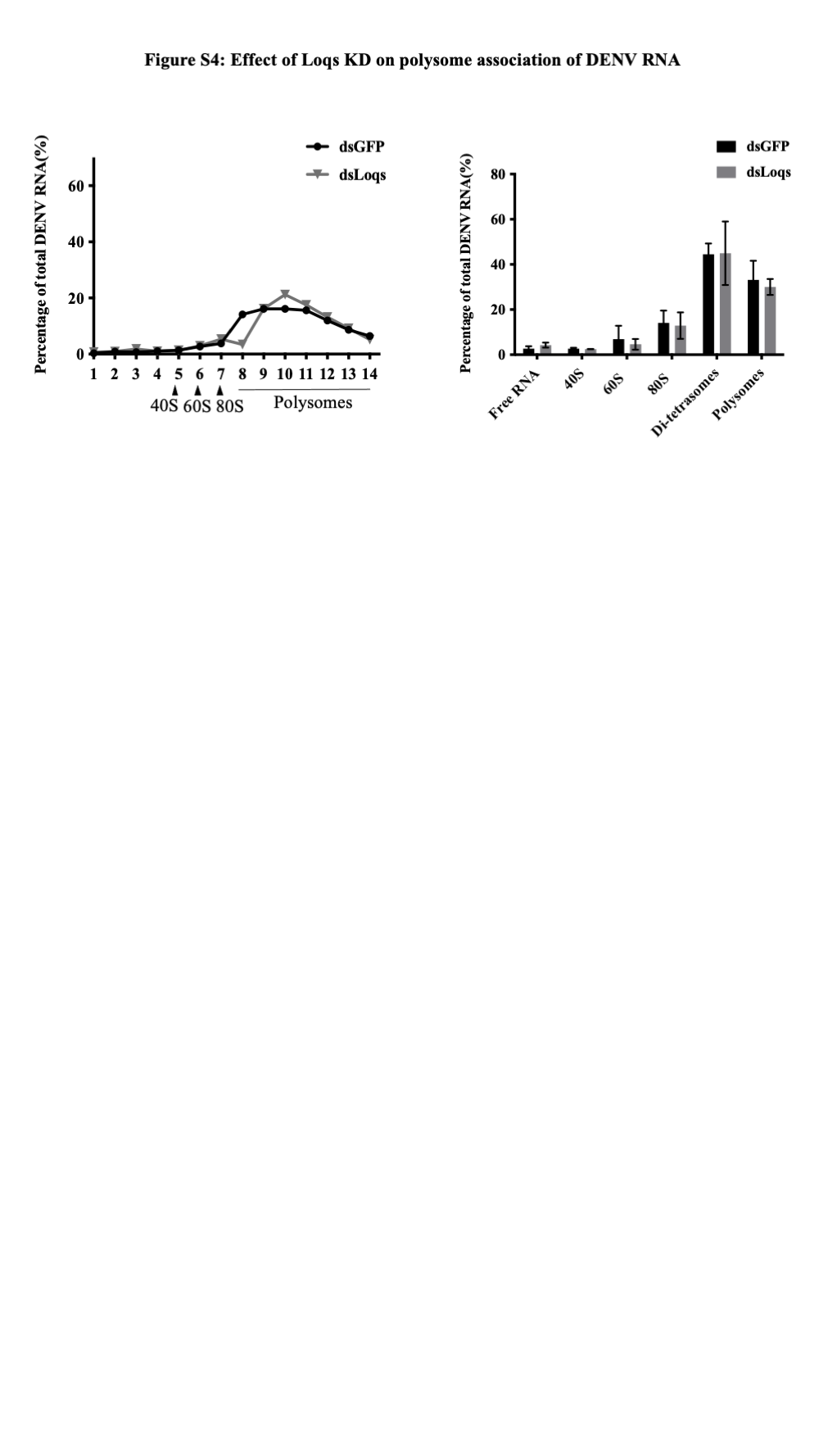

Supplement: S4 Fig — (A) DENV2 RNA abundance in each polysome fraction was measured by RT-qPCR and plotted as a percentage of the total RNA. A representative graph from three independent experiments is shown. (B) DENV2 RNA abundance in indicated polysome fractions plotted as an average from three independent experiments. (TIF) [file ppat.1010163.s004.tif]

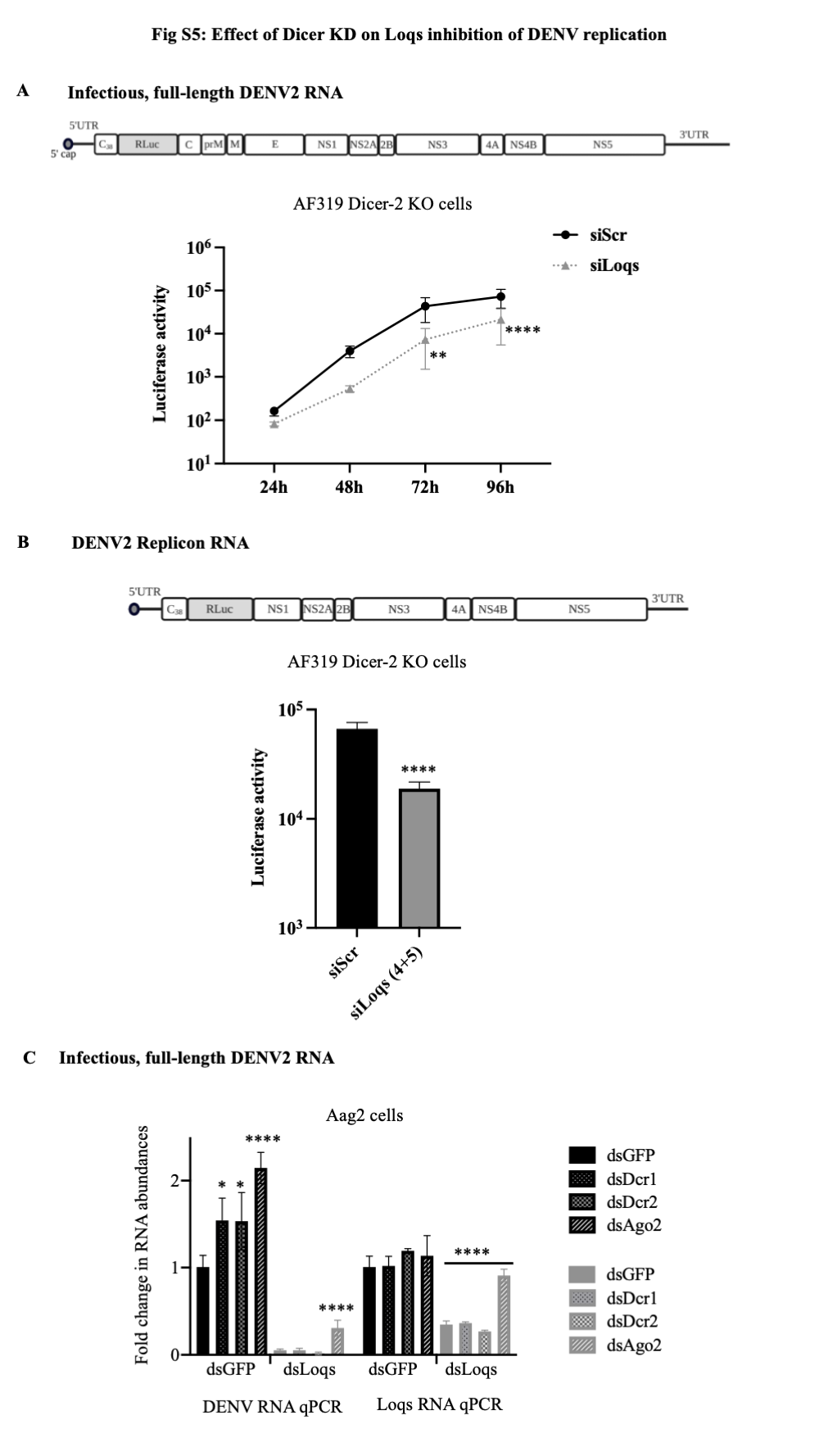

Supplement: S5 Fig — (A) AF319 Dicer-2 knock-out (KO) cells were transfected with the indicated siRNAs. 24 hrs after siRNA transfection, cells were infected with luciferase expressing DENV2 virus. Luciferase expression in cell lysates was measured at the indicated time points and represented as an average from three independent experiments (**p<0.005, ****p<0.0001). (B) AF319 Dicer-2 KO cells were transfected with the indicated siRNAs at a final concentration of 50nM (siLoqs-4 and siLoqs-5 were used together at a final concentration of 25nM each). At 24 hrs after siRNA transfection, cells were transfected with luciferase expressing DENV2-NGC replicon RNAs. Luciferase expression in cell lysates was measured 96 hrs post transfection (n = 4, ****p<0.0001). (C) Aag2 cells were transfected with the indicated dsRNAs. 24h after dsRNA transfection, cells were infected with DENV2 at a MOI of 0.1 for 96h. DENV and Loqs RNA abundances in the infected samples were measured by RT-qPCR and plotted as fold change over treatment with dsGFP. Data was normalized to internal control RPL32 mRNA levels (n = 3, *p<0.05, ****p<0.0001). (TIFF) [file ppat.1010163.s005.tiff]
