# Supplementary material for: Loquacious modulates flaviviral RNA replication in mosquito cells
Source: PLoS Pathog. 2022 Apr 28;18(4):e1010163. doi: 10.1371/journal.ppat.1010163 (PMC9089905; doi:10.1371/journal.ppat.1010163)
Supplement: S2 Table — List of primers used in cloning, RT-PCR and depletion analyses are shown. (DOC) [file ppat.1010163.s007.doc]

**Primers used in the study**

| **qPCR primers** | **Primer sequence (5’-3’)** |
| --- | --- |
| DENV universal FP | GGTTAGAGGAGACCCCTCCC |
| DENV universal RP | GGTCTCCTCTAACCTCTAGTCC |
| Loqs FP | AGATGGAAGTTGGACTGCCG |
| Loqs RP | TGATCTGGTTCGGTTCCAGC |
| Loqs PA exon-junction RP | CATGTGACCTCTTCATTG |
| Loqs PB exon-junction FP | GGCAATGAAGAGCTG |
| RPL32 FP | CAGTCCGATCGCTATGACAA |
| RPL32 RP | ATCATCAGCACCTCCAGCTC |
| Sec61A1 FP | TCGCGAGAGCTCAATGATCC |
| Sec61A1 RP | GCTCTGCTCCTTGACGAAGA |
| CHIKV FP | CATGCCGTCACAGTTAAGGA |
| CHIKV RP | AGACGTCGCCTTTGTACACC |
| WNV FP | GCTTTGCCACCTCTCTTCAC |
| WNV RP | CGGTTGATGGTTTCCACTCT |
| YFV FP | GAAATGCCTGCCCTTTATGA |
| YFV RP | GCACATGGCAACAGAAGCTA |
| ZIKV FP | ACCATACGGCCAACAAAGAG |
| ZIKV RP | TCCACAGCCAGGAAGAGACT |
| Dcr1 FP | GCTTCCGAGTGACACCTTCA |
| Dcr1 RP | TATGCAGAACACGACACGCT |
| Dcr2 FP | TGTGTCACAACTACCAATTCCCT |
| Dcr2 RP | CGTGTGATTCTCCAAACAGCC |
| Ago2 FP | GGCTGCTCACCCAATGTATCAAGA |
| Ago2 RP | AACCGTTCGTTTTGGCGTTGAT |

| **Cloning primers** | **Primer sequence (5’-3’)** |
| --- | --- |
| KF-DENV 3’UTR FP | GCTTGGCCCTGAAAAAGGGCAAGCTTGGCCCTGAAAAAGGGCAAAGCTCTTTAGAAAGCAAAACTTAAGATGAAAC |
| KF-DENV 3’UTR RP | AGCTGCCCTTTTTCAGGGCAAGAGCTGCCCTTTTTCAGGGCAAGAAGAGCTAGAACCTGTTGATTCAACAGC |
| KF-DENV-184 FP | GCTTGGCCCTGAAAAAGGGCAAGCTTGGCCCTGAAAAAGGGCAAAGCTCTTGGGAGGCCACAAACC |
| BG34-BamHI-BaSu-FP | ACAACCAAGTGACCGCGGATCTAGATCTGCATGAACGCACGAACACGAC |
| BG34-BaSu RP | TGTACGTGAGCGTACTTTTTTTGTTTTTTGTCACCTAAGGCTGATGTCCG |
| BG34-BamHI-BoxB-FP | ACAACCAAGTGACCGCGGATCTAGATCTGCATGGTGAGCAAGGGCGAGGAGCTGTTCA |
| BG34-NotI-BoxB-RP | TGTACGTGAGCGTACTTTTTTTGTTTTTTGGCGGGGAGGCGGCCCAAAGGGAGATCCGAC |
| pUB-NdeI EGFP FP | GGAATTCCATATGGTGAGCAAGGGCGAGGAGCTGT |
| pUB-SalI EGFP RP | ACGCGTCGACTCGGCATGGACGAGCTGTAC |
| pIEX4F sequencing primer | TGTTGGATATTGTTTCAG |
| pUB-5’ sequencing primer | ATTACTCAAGCGTTTCCTCGT |
| pUB-3’ sequencing primer | CTCTACAAATGTGGTATGGC |

| **siRNA** | **siRNA sense and antisense sequences (5’-3’)** |
| --- | --- |
| siScr | Sense: ACGUGACGUUCGGAGAAUUdTdT  Antisense: AAUUCUCCGAACGUCACGUdTdT |
| siSec61A1-1 | Sense: GCAUAAAAUUCCUGGAAAUdTdT  Antisense: AUUUCCAGGAAUUUUAUGCdTdT |
| siSec61A1-3 | Sense: CACACAUAGUACUGUUUAAdTdT  Antisense: UUAAACAGUACUAUGUGUGdTdT |
| siLoqs-2 | Sense: GUUCGGGUGGCGAAGAAAUdTdT  Antisense: AUUUCUUCGCCACCCGAACdTdT |
| siLoqs-4 | Sense: GAGCAAGAAGAUUGCCAAAdTdT  Antisense: UUUGGCAAUCUUCUUGCUCdTdT |
| siLoqs-5 | Sense: CUCGCAACUCGCUGGAAUAdTdT  Antisense: UAUUCCAGCGAGUUGCGAGdTd |
| siLoqs 3’-1 | Sense: GUUACACCAUUUCUCAAUUdTdT  Antisense: AAUUGAGAAAUGGUGUAACdTdT |
| siLoqs 3’-2 | Sense: GUGCAACGAAAAUCAAUUUdTdT  Antisense: AAAUUGAUUUUCGUUGCACdTdT |
| siSec61A1 | Sense: GCAUAAAAUUCCUGGAAAUdTdT  Antisense: AUUUCCAGGAAUUUUAUGCdTdT |
| siDENV-1 | Sense: UGCUGAAACGCGAGAGAAAdTdT  Antisense: UUUCUCUCGCGUUUCAGCAdTdT |
| siDENV-2 | Sense: CCAAAGAGGUAGUGGACAAdTdT  Antisense: UUGUCCACUACCUCUUUGGdTdT |

| **dsRNA** | **Primer sequences with T7 promoter (5’-3’)** |
| --- | --- |
| dsEGFP FP | taatacgactcactatagggATGGTGAGCAAGGGCGAGGAGC |
| dsEGFP RP | aatacgactcactatagggTCTTGAAGTTCACCTTGATGCCGTT |
| dsSec61A1 FP | taatacgactcactatagggAGGACCGGGCTCTGTTTAAT |
| dsSec61A1 RP | taatacgactcactatagggATATGACCACCGCAAAGACC |
| dsLoqs FP | taatacgactcactatagggACGGTTTGAGGTGACCTACGTTGACATTGAC |
| dsLoqs RP | taatacgactcactatagggGTTGTTGGCGGGAGATGGTGTTCCACAAT |
| dsLoqs-PB-FP | taatacgactcactatagggCTGAAGGCGGCCAGCATTTCCGGGCGG |
| dsLoqs-PB-RP | taatacgactcactatagggCTGCAGTTGGCGCAGAGTTTCG |
| dsDcr1-FP | taatacgactcactatagggATGGCTTACCATTGGACGG |
| dsDcr1-RP | taatacgactcactatagggGCTTCTCCCGGCATACATT |
| dsDcr2 FP | taatacgactcactataggggCATTGACGACGAAATCATCGTC |
| dsDcr2 RP | taatacgactcactatagggCCATGGCATCCGCCGGTGTCT |
| dsAgo2 FP | taatacgactcactatagggCGGCTGCTAGTGTCCAACAG |
| dsAgo2 RP | taatacgactcactatagggACCGTCCTGTATCTCGGTCC |
